# Do patients’ and referral centers’ characteristics influence multiple sclerosis phenotypes? Results from the Italian multiple sclerosis and related disorders register

**DOI:** 10.1007/s10072-022-06169-7

**Published:** 2022-06-07

**Authors:** Roberto Bergamaschi, Ettore Beghi, Cristina Bosetti, Michela Ponzio, Claudia Santucci, Vito Lepore, Paola Mosconi, on behalf of the Italian Multiple Sclerosis and Related Disorders Register Centers Group and the Scientific Committee of Italian SM and Related Disorders Register, U. Aguglia, M. P. Amato, A. L. Ancona, B. Ardito, C. Avolio, R. Balgera, P. Banfi, V. Barcella, P. Barone, P. Bellantonio, A. Berardinelli, R. Bergamaschi, P. Bertora, M. Bianchi, P. Bramanti, V. Brescia Morra, G. Brichetto, A. M. Brioschi, M. Buccafusca, S. Bucello, V. Busillo, B. Calchetti, R. Cantello, M. Capobianco, F. Capone, L. Capone, D. Cargnelutti, M. Carrozzi, E. Cartechini, G. Cavaletti, P. Cavalla, M. G. Celani, R. Clerici, M. Clerico, E. Cocco, P. Confalonieri, M. G. Coniglio, A. Conte, F. Corea, S. Cottone, P. Crociani, F. D’Andrea, M. C. Danni, G. De Luca, D. de Pascalis, M. De Riz, F. De Robertis, G. De Rosa, N. De Stefano, M. Della Corte, A. Di Sapio, R. Docimo, M. Falcini, N. Falcone, S. Fermi, E. Ferraro, M. T. Ferrò, M. Fortunato, M. Foschi, A. Gajofatto, A. Gallo, P. Gallo, M. Gatto, P. Gazzola, A. Giordano, F. Granella, M. F. Grasso, M. G. Grasso, L. M. E. Grimaldi, P. Iaffaldano, D. Imperiale, M. Inglese, R. Iodice, S. Leva, V. Luezzi, A. Lugaresi, G. Lus, D. Maimone, L. Mancinelli, G. T. Maniscalco, G. A. Marfia, B. Marini, A. Marson, N. Mascoli, L. Massacesi, F. Melani, M. Merello, G. Meucci, M. Mirabella, S. Montepietra, D. Nasuelli, P. Nicolao, F. Passantino, F. Patti, M. Peresson, I. Pesci, C. Piantadosi, M. L. Piras, M. Pizzorno, K. Plewnia, C. Pozzilli, A. Protti, R. Quatrale, S. Realmuto, G. Ribizzi, S. Rinalduzzi, A. Rini, S. Romano, M. Romeo, M. Ronzoni, P. Rossi, M. Rovaris, G. Salemi, G. Santangelo, M. Santangelo, G. Santuccio, P. Sarchielli, L. Sinisi, P. Sola, C. Solaro, D. Spitaleri, S. Strumia, T. Tassinari, S. Tonietti, C. Tortorella, R. Totaro, A. Tozzo, G. Trivelli, M. Ulivelli, P. Valentino, S. Venturi, M. Vianello, M. Zaffaroni, R. Zarbo, Maria Trojano, Mario Alberto Battaglia, Marco Capobianco, Maura Pugliatti, Monica Ulivelli, Paola Mosconi, Claudio Gasperini, Francesco Patti, Maria Pia Amato, Roberto Bergamaschi, Giancarlo Comi

**Affiliations:** 1 IRCCS Fondazione Mondino, Pavia, Italy; 2 Istituto di Ricerche Farmacologiche Mario Negri IRCCS, Milan, Italy; 3 Scientific Research Area, Italian Multiple Sclerosis Foundation, Genoa, Italy; 4 Department of Clinical Sciences and Community Health, University of Milan, Milan, Italy; 5 Laboratorio di Ricerca per il Coinvolgimento dei Cittadini in Sanità, Dipartimento di Salute Pubblica, Istituto di Ricerche Farmacologiche Mario Negri IRCCS, Via Mario Negri 2, 20156 Milan, Italy

**Keywords:** Multiple sclerosis phenotypes, Centers’ characteristics, Real-world data, Italian Multiple Sclerosis Register

## Abstract

**Background:**

Multiple sclerosis (MS) is characterized by phenotypical heterogeneity, partly resulting from demographic and environmental risk factors. Socio-economic factors and the characteristics of local MS facilities might also play a part.

**Methods:**

This study included patients with a confirmed MS diagnosis enrolled in the Italian MS and Related Disorders Register in 2000–2021. Patients at first visit were classified as having a clinically isolated syndrome (CIS), relapsing–remitting (RR), primary progressive (PP), progressive-relapsing (PR), or secondary progressive MS (SP). Demographic and clinical characteristics were analyzed, with centers’ characteristics, geographic macro-areas, and Deprivation Index. We computed the odds ratios (OR) for CIS, PP/PR, and SP phenotypes, compared to the RR, using multivariate, multinomial, mixed effects logistic regression models.

**Results:**

In all 35,243 patients from 106 centers were included. The OR of presenting more advanced MS phenotypes than the RR phenotype at first visit significantly diminished in relation to calendar period. Females were at a significantly lower risk of a PP/PR or SP phenotype. Older age was associated with CIS, PP/PR, and SP. The risk of a longer interval between disease onset and first visit was lower for the CIS phenotype, but higher for PP/PR and SP. The probability of SP at first visit was greater in the South of Italy.

**Discussion:**

Differences in the phenotype of MS patients first seen in Italian centers can be only partly explained by differences in the centers’ characteristics. The demographic and socio-economic characteristics of MS patients seem to be the main determinants of the phenotypes at first referral.

**Supplementary Information:**

The online version contains supplementary material available at 10.1007/s10072-022-06169-7.

## Introduction

Multiple sclerosis (MS) is a chronic immune-mediated neurological disease affecting more than two million people in the world [1, 2]. In Italy, around 129,220 people are estimated to live with MS, with a prevalence of about 210 cases per 100,000 inhabitants, with the exception of Sardinia, where the prevalence is higher (390 cases per 100,000) [3]. MS is characterized by five major phenotypes, namely the clinically isolated syndrome (CIS), relapsing–remitting (RR), primary progressive (PP), progressive-relapsing (PR), and secondary progressive MS (SP) [4, 5]. The same risk factors, i.e., genetic patterns, tobacco smoking, exposure to air pollutants, viral infections, low vitamin D levels, and juvenile obesity [6, 7], also affect the type and severity of the disease. Other factors unrelated to demographic and clinical features, like the variability of contacts, diagnostic paths, and therapeutic capabilities, may also influence the MS phenotypes. A wide range of services and treatments are offered to people with MS, varying based on MS centers’ characteristics, geographic distribution, and socioeconomic conditions of the local populations [8].

Real-world observational studies, such as registers, provide important information on the history, course, and severity of MS, useful for public health aims and to complement clinical trial results. Increasing numbers of MS registers have been established in recent years, providing good opportunities for advance in MS research, pharmacovigilance, and sharing data among international MS data repositories [9, 10]. In Italy, the AISM (Associazione Italiana Sclerosi Multipla) — a powerful patients’ organization founded in 1968 — advocates for the rights of people with MS, supporting a network of local branches who collaborate with healthcare professionals and clinical centers. AISM endorsed the establishment of the Italian MS and Related Disorders Register which now includes a large cohort of people with MS and provides a descriptive picture of the baseline demographic and clinical characteristics of the disease, facilitating comparisons of socio-demographic, geographic, and clinical factors [11].

This study describes MS phenotypes at first visit, comparing differences in MS phenotypes across centers’ characteristics and location, and patients’ socio-demographic and clinical variables.

## Material and methods

### Data source

Data used for the present analysis were extracted from the Italian MS and Related Disorders Register, a nationwide database, sustained by a network of 161 MS centers and promoted in 2014, in continuity with the existing Italian MSDatabase Network [12]. The Italian Register now contains data on more than 72,000 exclusively registered patients, enrolled using a common minimum dataset, and this sample size represents approximately the 56% of Italian MS patients. During its first years, the Italian Register data were stored on a client server (iMed© software), but since 2017, a web-based tool has been developed, onto which all data have been gradually transferred (http://www.registroitalianosm.it). Data are centrally monitored to guarantee the quality of information and centers are periodically contacted with ad hoc queries on missing data or inconsistencies in the variables recorded. Data of the Italian Register are required to be updated every 6 months [11].

For the present study, we also retrieved information on the centers included in the Italian Register through a survey conducted in 2020. In case of missing information, the survey results were integrated with data from the 2018 Barometer of MS — a benchmarking tool published by AISM, to provide an accurate picture of MS management across health and social care systems in Italy [13]. The survey collected data on the type of center: organizational structure of the operating unit, number of patients treated, number of neurologists and nurses dedicated to MS patients, difficulties in access to disease-modifying treatments (DMT), beds dedicated to MS patients, and presence of formally recognized Diagnostic and Critical Pathways (Percorsi Diagnostico-Terapeutici Assistenziali — PDTA). We classified the areas where centers were located into five macro-areas, i.e., North-West (Piedmont, Valle d’Aosta, Liguria, Lombardy), North-East (Trentino-Alto Adige, Veneto, Friuli-Venezia Giulia, Emilia-Romagna), Center (Tuscany, Umbria, Marche, Lazio), South (Abruzzo, Molise, Campania, Puglia, Basilicata, Calabria), and Islands (Sicily, Sardinia). We assigned to each center the quintile of deprivation index (DI) of the corresponding province, updated and revised on the basis of 2011 Italian census data [14, 15]. This index measures social and material deprivation in the presence of low educational level, unemployment, living in rental property, living in a crowded house, and living in a single-parent family. The DI was then grouped in three classes, I–II (low deprivation), III (moderate deprivation), and IV–V (high deprivation).

### Study population

The study population is composed of all consecutive patients with a confirmed diagnosis of MS (according to McDonald 2001 criteria, and subsequent updates of 2010 and 2017 [16]) enrolled in 2000–2021. The MS phenotypes were defined through an algorithm, developed and validated according to Scientific Committee, considering date at onset, diagnosis, progression start, and relapse, together with the information reporting whether or not the onset of progression coincided with the date of onset. Dates were ordered from the earliest to the latest and, considering the temporal sequence, five disease phenotypes were so defined: CIS, PP, PR, RR, SP.

### Statistical analysis

Baseline characteristics are presented using summary statistics, as appropriate. We computed the odds ratio (OR) and corresponding 95% confidence interval (CI) for CIS, PP/PR, and SP phenotypes compared to the RR phenotype according to selected patients’ and centers’ characteristics, using univariate and multivariate, multinomial, multilevel logistic regression models including random effects for center. These models allowed to take into account for the hierarchical nature of the data (i.e., patients and clinicians are nested within hospitals). We also performed models including a further level for geographic regions that provided very similar results. Multivariate models were adjusted for age, sex, and time between disease onset and first visit. For statistical analyses we used the software SAS, version 9.4 (SAS Institute, Cary, NC, USA).

## Results

As of 31 May 2021, there were 72,283 patients in the Italian Register. We first excluded 30,166 patients enrolled before 2000 or with enrolment date missing and those not included in the minimum dataset (i.e., with neuromyelitis optic spectrum disorder or no McDonald-confirmed MS diagnosis, and incomplete/inconsistent information on relevant variables). We also excluded patients with only one visit, and those from centers that did not participate in the survey. A total of 35,243 patients from 106 centers were thus included in the present study (Supplementary Fig. 1).

Table 1 reports the baseline characteristics of the patients analyzed: two-third were female (67%); mean age was 33.4 years at the onset, and 36.2 years at the first visit; a median of 13 months passed between onset and first visit; about 80% had only one symptom at onset; median EDSS score at first visit was 2.4, with a median MS disease severity index of 4.7; most patients received an RR diagnosis, and about a quarter had a CIS diagnosis. The distribution of 5437 patients enrolled from 2000 and included in the minimum dataset (i.e., with a confirmed MS diagnosis and no incomplete/inconsistent information on relevant variables regarding diagnosis and visits) but not considered in the final cohort is presented in Supplementary Table 1. As compared to those included in our analyses, those patients were more frequently enrolled in 2015–2021, had a higher time between disease onset and first visit but a lower time between disease onset and diagnosis, and were more frequently under a DMT treatment.

**Table 1:** Baseline characteristics of 35,243 multiple sclerosis patients in the Italian MS and Related Disorders Register, 2000–2021

	Patients
Calendar year at first visit — No. (%)	
2000–2009	10,887 (30.9)
2010–2014	10,638 (30.2)
2015–2021	13,718 (38.9)
Sex — No. (%)	
Female	23,515 (66.7)
Age at onset (years)	
Mean (SD)	33.4 (11.1)
Age at first visit (years)	
Mean (SD)	36.2 (11.7)
Time between disease onset and first visit (months)	
Median (IQR)	13.0 (3.0–33.6)
No. of symptoms at onset —No. (%)	
1	28,341 (80.4)
≥ 2	6,144 (17.4)
Symptoms at onset^a^ — No. (%)	
Brainstem	9,245 (26.2)
Optic	7,960 (22.6)
Supratentorial	9,972 (28.3)
Spinal	11,061 (31.4)
Age at diagnosis (years)	
Mean (SD)	35.1 (11.7)
Time between disease onset and diagnosis (months)	
Median (IQR)	9.0 (2.6–28.0)
First disease phenotype — No. (%)	
Clinically isolated syndrome	33,473 (95.0)
Primary progressive	1,770 (5.0)
Disease phenotype at first visit^b^ — No. (%)	
Clinically isolated syndrome	9,310 (26.4)
Relapsing–remitting	23,588 (66.9)
Primary progressive	1,395 (4.0)
Progressive-relapsing	375 (1.1)
Secondary progressive	575 (1.6)
Type of DMT at first visit — No. (%)	
No treatment	24,273 (68.9)
First-line treatment	9,461 (26.8)
Second-line treatment	821 (2.3)
Off-label treatment	688 (1.9)
EDSS score at first visit	
Median (IQR)	2.0 (1.0–3.0)
MS severity at first visit	
Median (IQR)	4.7 (2.4–6.6)

*DMT*, disease-modifying therapy; *EDSS*, Expanded Disability Status Scale; *IQR*, interquartile range; *SD*, standard deviation. ^a^Not mutually exclusive. ^b^Defined as the last of the three disease courses recorded before the first visit to a center in the Register

**Fig. 1:**
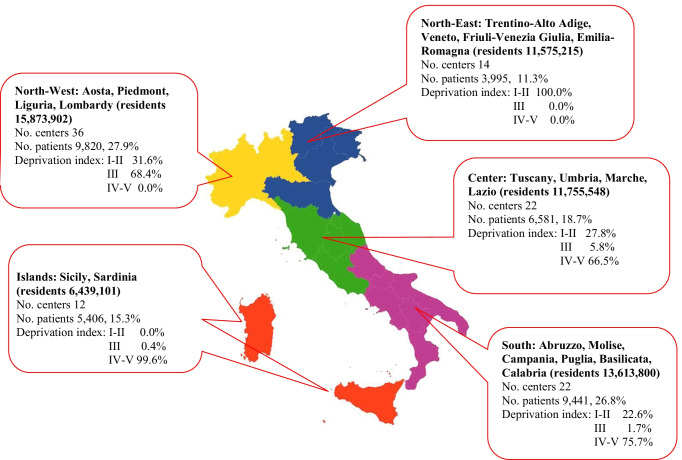
Distribution of study centers from Italian MS and Related Disorders Register by geographic macro-area at first visit and deprivation index

Figure 1 shows the distribution of the study centers by geographic macro-areas, with the corresponding DI. More than half of the patients were attending North-West and South centers, where there were more participating centers (36 and 22, respectively). Centers in the South were in more deprived areas than North-West centers. About 19% of patients were in the Center and 15% in the Islands, both with a high DI. Finally, 11% of patients were attending North-East centers, where the DI was lowest. Supplementary Table 2 illustrates the characteristics of the 106 centers.

The disease phenotypes at first visit varied with patients and centers’ characteristics (Table 2). Females were less frequent among PP/PR and SP patients; age at first visit was older in PP/PR and SP patients. Time between disease onset and first visit was shorter for CIS, but longer for PP/PR and SP patients. The disease phenotypes were similar according to the centers’ characteristics, as geographical macro-areas (except for larger proportion of PP/PR phenotypes in the Islands and a smaller proportion in the South), number of patients followed, healthcare professionals dedicated, MS-dedicated beds, and access to DMT.

**Table 2:** Distribution of disease phenotypes at first visit.^a^ according to selected patients’ and center characteristics among 35,243 patients in the Italian MS and Related Disorders Register, 2000–2021

	Total (35,243)	Clinically isolated syndrome (9,310)	Relapsing–remitting (23,588)	Primary progressive/progressive-relapsing (1,770)	Secondary progressive (575)
	No. (%)	No. (%)	No. (%)	No. (%)	No. (%)
Patients’ characteristics					
Year at first visit					
2000–2009	10,887 (30.9)	3,098 (33.3)	7,158 (30.4)	501 (28.3)	130 (22.6)
2010–2014	10,638 (30.2)	2,719 (29.1)	7,197 (30.5)	500 (28.3)	222 (38.6)
2015–2021	13,718 (38.9)	3,493 (37.5)	9,233 (39.1)	769 (43.5)	223 (38.8)
Sex					
Female	23,515 (66.7)	6,313 (67.8)	15,955 (67.6)	912 (51.5)	335 (41.7)
Age at first visit					
< 35 years	17,032 (48.3)	5,068 (54.4)	1,1732 (49.7)	164 (9.3)	68 (11.8)
≥ 35 years	18,211 (51.7)	4,242 (45.6)	1,1856 (50.3)	1,606 (90.7)	507 (88.2)
Time between disease onset and first visit					
< 13 months	17,605 (50.0)	7,937 (85.3)	9,257 (39.2)	388 (21.9)	23 (4.0)
≥ 13 months	17,638 (50.0)	1,373 (14.8)	14,331 (60.8)	1,382 (78.1)	552 (96.0)
Centers’ characteristics					
Area of first visit					
North-West	9,820 (27.9)	2,036 (21.9)	7,201 (30.5)	436 (24.6)	147 (25.6)
North-East	3,995 (11.3)	1,200 (12.9)	2,449 (10.4)	267 (15.1)	79 (13.7)
Center	6,581 (18.7)	1,780 (19.2)	4,442 (18.8)	255 (14.4)	104 (18.1)
South	9,441 (26.8)	2,815 (30.2)	5,961 (25.3)	469 (26.5)	196 (34.1)
Islands	5,406 (15.3)	1,479 (15.9)	3,535 (15.0)	343 (19.4)	49 (8.5)
Deprivation index					
I–II	11,060 (31.4)	3,310 (35.6)	7,006 (29.7)	601 (34.0)	143 (24.9)
III	7,281 (20.7)	1,354 (14.5)	5,455 (23.1)	335 (18.9)	137 (23.8)
IV–V	16,902 (48.0)	4,646 (49.9)	11,127 (47.2)	834 (47.1)	295 (51.3)
Number of patients with multiple sclerosis followed at the center					
< 500	5,187 (14.7)	1,575 (16.9)	3,246 (13.8)	261 (14.8)	105 (18.3)
≥ 500	30,056 (85.3)	7,735 (83.1)	20,342 (86.2)	1,509 (85.3)	470 (81.7)
Number of neurologists dedicated to multiple sclerosis^b^					
< 3	4,271 (12.2)	1,175 (12.6)	2,766 (11.7)	234 (13.2)	96 (16.7)
≥ 3	30,735 (87.2)	8,091 (86.9)	20,649 (87.5)	1,524 (86.1)	471 (81.9)
Number of nurses dedicated to multiple sclerosis^b^					
< 2	1,813 (5.1)	525 (5.6)	1,152 (4.9)	92 (5.2)	44 (7.7)
≥ 2	29,241 (83.0)	8,041 (86.4)	19,298 (81.8)	1,477 (83.5)	425 (73.9)
Centers with difficulties in access to DMT^b^					
No	16,196 (46.0)	3,688 (39.6)	11,537 (48.9)	673 (38.0)	298 (51.8)
Yes	18,767 (53.3)	5,553 (59.7)	11,855 (50.3)	1,084 (61.2)	275 (47.8)
Hospital beds dedicated to multiple sclerosis					
No	19,988 (56.7)	5,799 (62.3)	12,797 (54.3)	1,077 (60.9)	315 (54.8)
Yes	15,255 (43.3)	3,511 (37.7)	10,791 (45.8)	693 (39.2)	260 (45.2)
PDTA^b^					
No	5,093 (14.5)	1,530 (16.4)	3,154 (13.4)	290 (16.4)	119 (20.7)
Yes	29,988 (85.1)	7,762 (83.4)	20,298 (86.1)	1,476 (83.4)	452 (78.6)

*DMT*, disease-modifying therapy; *PDTA*, Diagnostic and Critical Pathways. ^a^Defined as the last among the three disease courses registered before the first visit at a center of the register. ^b^Discrepancies in the total are due to missing values

Table 3 gives the results of multivariate analysis for CIS, PP/PR, and SP phenotypes at first visit compared to RR phenotypes, according to selected patients’ and centers’ characteristics. The corresponding results of univariate analyses are given in Supplementary Table 3. The OR of presenting more advanced MS phenotypes, compared to the RR phenotype, significantly diminished over the years (OR = 0.74 of PP/PR for 2010–2014 and 2015–2021 vs 2000–2009, and OR = 0.50 of SP for 2015–2021 vs 2000–2009). Females were at significantly lower risk than males of PP/PR (OR = 0.47) or SP phenotype (OR = 0.62). Older age at first visit was associated with the risk of CIS (OR = 1.37 for ≥ 35 vs < 35 years), and particularly of PP/PR (OR = 9.21) and SP (OR = 4.53). The risk of a long interval between disease onset and first visit was lower for CIS phenotype (OR = 0.11 for ≥ 13 vs < 13 months), but higher for PP/PR (OR = 1.49) and particularly for SP (OR = 10.19). The probability of an SP phenotype was greater in the South (OR = 1.86 vs North-West) and a CIS in the North-East (OR = 1.64). No meaningful associations with MS phenotype were found for other centers’ characteristics or DI.

**Table 3:** Multivariate odds ratios (OR) and corresponding confidence intervals (CI) for clinically isolated syndrome (CIS), primary progressive (PP)/progressive-relapsing (PR), and secondary progressive (SP) phenotypes at first visit compared to relapsing–remitting (RR) phenotypes according to selected patients’ and center characteristics among 35,243 patients in the Italian MS and Related Disorders Register, 2000–2021

	CIS (9,310)	PP/PR (1,770)	SP (575)
	OR (95% CI)^a^	OR (95% CI)^a^	OR (95% CI)^a^
Patients’ characteristics			
Calendar year at first visit			
2000–2009	1^b^	1^b^	1^b^
2010–2014	1.00 (0.93–1.08)	**0.74 (0.65–0.86)**	0.83 (0.65–1.05)
2015–2021	1.00 (0.93–1.08)	**0.74 (0.65–0.85)**	**0.50 (0.39–0.65)**
Sex			
Male	1^b^	1^b^	1^b^
Female	1.01 (0.95–1.08)	**0.47 (0.42–0.52)**	**0.62 (0.52–0.74)**
Age at first visit			
< 35 years	1^b^	1^b^	1^b^
≥ 35 years	**1.37 (1.29–1.46)**	**9.21 (7.80–10.87)**	**4.53 (3.49–5.88)**
Time between disease onset and first visit			
< 13 months	1^b^	1^b^	1^b^
≥ 13 months	**0.11 (0.10–0.11)**	**1.49 (1.32–1.69)**	**10.19 (6.69–15.52)**
Center characteristics			
Area of first visit			
North-West	1^b^	1^b^	1^b^
North-East	**1.64 (1.03–2.59)**	1.44 (0.87–2.39)	1.50 (0.88–2.56)
Center	1.48 (0.98–2.23)	1.09 (0.69–1.71)	1.30 (0.80–2.10)
South	1.39 (0.93–2.07)	1.51 (0.98–2.34)	**1.86 (1.18–2.95)**
Islands	0.80 (0.47–1.36)	1.36 (0.77–2.38)	0.86 (0.46–1.59)
Deprivation index			
I–II	1^b^	1^b^	1^b^
III	0.74 (0.49–1.10)	0.72 (0.47–1.12)	1.15 (0.70–1.87)
IV–V	0.82 (0.59–1.16)	1.00 (0.70–1.44)	1.44 (0.96–2.16)
No. patients with multiple sclerosis followed at the center			
< 500	1^b^	1^b^	1^b^
≥ 500	0.86 (0.63–1.16)	1.15 (0.82–1.62)	0.85 (0.58–1.24)
Number of neurologists dedicated to multiple sclerosis			
< 3	1^b^	1^b^	1^b^
≥ 3	1.09 (0.78–1.54)	1.17 (0.79–1.71)	0.94 (0.62–1.45)
Number of nurses dedicated to multiple sclerosis			
< 2	1^b^	1^b^	1^b^
≥ 2	1.13 (0.71–1.80)	1.64 (0.91–2.95)	0.93 (0.49–1.78)
Center with difficulties in access to DMT			
No	1^b^	1^b^	1^b^
Yes	1.26 (0.93–1.70)	1.33 (0.96–1.84)	1.12 (0.78–1.62)
Hospital beds dedicated to multiple sclerosis patients			
No	1^b^	1^b^	1^b^
Yes	0.99 (0.72–1.35)	0.92 (0.65–1.29)	1.11 (0.77–1.60)
Presence of a PDTA			
No	1^b^	1^b^	1^b^
Yes	1.12 (0.79–1.60)	1.17 (0.79–1.72)	0.66 (0.44–0.99)

*DMT*, disease-modifying therapy; *PDTA*, Diagnostic and Critical Pathways. ^a^Odds ratio from multinomial, multilevel logistic regression models with random effects for center compared to RR phenotype adjusted for age, sex, and time between disease onset and first visit. ^b^Reference category

In boldface statistical significant estimates

## Discussion

This retrospective study of a large cohort of MS patients depicts the distribution of MS phenotypes at first visit comparing different geographic macro-areas, patients’ socio-demographic features, and the characteristics of referral centers. The demographic and socio-economic characteristics of MS patients seem to be the main determinants of the phenotypes at first referral, while differences in the phenotypes can be only partly explained by differences in the centers’ structures, capabilities, and patient loads.

PP/PR and SP patients were older at first visit than CIS ones. This agrees with previous studies reporting that these disease variants — whether or not following an RR course — tend to occur at an older age, preferably in men [17].

We found that presentation with advanced MS phenotype at referral centers tended to decline over time. This is not unexpected because patients are increasingly referred by local neurologists to an MS center where they can receive drugs that cannot be administered elsewhere. The time between disease onset and first visit was longer in PP/PR and SP phenotypes, and this is a diagnostic challenge for these patients, who are also older and more likely to be female. Greater attention is needed in intercepting these late-onset MS cases considering also socio-cultural and environmental factors in long MS [18, 19].

The characteristics of MS centers, such as the number of patients followed, number of neurologists and nurses, access to DMT, beds dedicated, and presence of a PDTA, did not appear related to the disease phenotype. This could be the result of a multidisciplinary approach (confirmed in the majority of centers) and the contribution of the AISM which prompts the collection of epidemiological data as well as continuous exchange of experience and information among centers [8]. Moreover, the long history of data sharing through the Italian Register, started in 2010, has favored a standardization of diagnostic and treatment practices among centers [11, 12]. Although formally incorporated within their respective hospitals, MS centers in Italy historically form part of a broader network committed to ensuring all people with MS receive the appropriate lengthy care. The network permits communication and supports high-quality care within and across regions and over time. It also helps contrast the disparities in health systems [20].

Regarding the availability of DMT, we are aware that more than 50% of the centers reported difficulties in the access, mainly depending on the different reimbursement policies that each regional health service establishes. This shows the importance of a data collected through a Register with a national representativeness, which can contribute to the debate on the reimbursement of new, more effective therapies even in the early stages of the disease. Moreover, the support of a large patient association involved in all the phases of the Register can lead to significant future improvements.

Our results show more CIS phenotypes in the North-East and Center and more SP in Southern regions. The predominance of CIS in the North can be explained by the prompter access of patients at the start of symptoms to one of the MS centers widely distributed over this area, leading them to earlier diagnosis of the disease. The larger number of SP MS in the South compared to the North of Italy is harder to explain, except that most patients with MS in the South are referred to the few centers of excellence, and the distribution of DI is different.

The study has some strengths. We investigated a large nationwide sample of people with MS. As patients are followed in tertiary centers, we expect that the diagnosis is correct and management of the disease is appropriate. Then, the network of centers in the Italian Register forms the basis for standardized and comparable data collection. Moreover, real-world data like that provided by the MS registers allow assessment of the heterogeneity of the setting, centers’ and patients’ characteristics within national and international geographic realities [21, 22].

However, the study also has some limitations. First, our Register does not represent all Italian centers where SM patients can be diagnosed and followed. All regions are represented in the Register but not all the MS centers. Indeed, North-West and Southern centers recorded about 40% of cases, leaving only 20% in the Center. Territorial distribution of Centers also varies in the Italian macro-areas and can be explained by the different rates of establishment and growth of new centers according to local priorities, as well as the development of some large referral centers in southern regions.

Second, selection bias cannot be excluded; however, the analysis of only MS patients enrolled since 2000 reduced the number of excluded cases and does not invalidate the results of the analyzed cohort. Patients excluded from the present analysis had indeed few differences compared to those included in the analysis (Supplementary Table 1). In addition, milder forms of the disease might not have been registered as managed by general neurologists in the community, and we do know that more severe MS forms are not followed in a hospital setting.

Third, the data quality remains one of the weak points of multicenter registers. We are addressing this issue by monitoring the centers every 2 months and sending out periodic detailed reports to the centers. A network of research assistants is engaged in more than 90 centers collaborating with local neurologists both in the input of new cases and in the update of data, especially as regards the historical cohort. Eight quality indicators have been developed and periodically communicated to the centers, and other 5 indicators are currently under revision and will soon be implemented (11). In conclusion, our findings indicate that differences in the phenotypes of people with MS who are seen first in Italian centers can be only partly explained by differences in the centers’ structures, capabilities, and patient loads. However, the socio-economic context of the local population might help explain differences in the proportions of patients at the two extremes of the disease spectrum. However, the overall picture is getting better, as shown by the trends of the disease variants from the old to the more recent periods. To the best of our knowledge, there are no studies where data deriving from MS registers have been specifically used to evaluate the possible relationships between the characteristics of the MS centers and the phenotype of the MS patients. We therefore agree with Magyari [9, 23] and Allen-Philbey [24] that population-based registries and integrated databases are crucial for an accurate description of both the changing prognosis of MS and the different characteristics of the various MS phenotypes.

## Supplementary Information

Below is the link to the electronic supplementary material.

Supplementary file1 (DOCX 140 kb)

## Data Availability

The dataset analyzed for this study is available from the corresponding author upon reasonable request.
