# Supplementary material for: Do patients’ and referral centers’ characteristics influence multiple sclerosis phenotypes? Results from the Italian multiple sclerosis and related disorders register
Source: Neurol Sci. 2022 Jun 7;43(9):5459–69. doi: 10.1007/s10072-022-06169-7 (PMC9385759; doi:10.1007/s10072-022-06169-7)
Supplement: Supplementary file 1 — Supplementary file1 (DOCX 140 kb) [file 10072_2022_6169_MOESM1_ESM.docx]

**Supplementary Figure 1.** Flowchart describing the selection from the original cohort of 72,283 people with MS to the cohort of 35,243 analyzed.


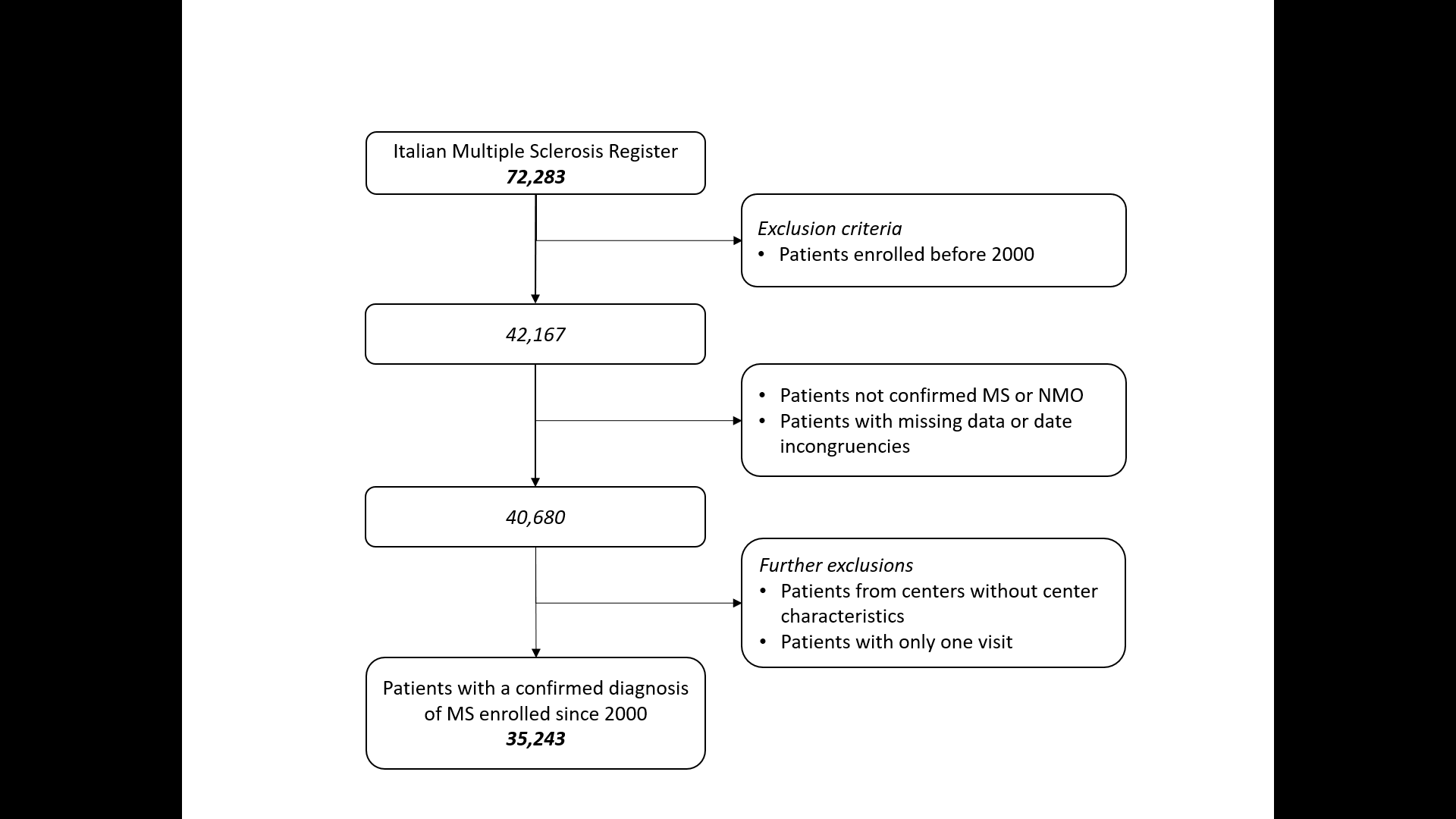


**Supplementary Table 1.** Baseline characteristics of 5,437 patients enrolled since 2000 and included in the minimum dataset from the Italian MS and Related Disorders Register, but not considered in the final study cohort.

| **Characteristics** | **Patients** |
| --- | --- |
|  |  |
| Calendar year at first visit **-** No. (%) |  |
| 2000-2009 | 1,215 (22.4) |
| 2010-2014 | 1,192 (21.9) |
| 2015-2021 | 3,030 (55.7) |
| Sex **-** No. (%) |  |
| Female | 3,725 (68.5) |
| Age at onset (years) |  |
| Mean (SD) | 34.5 (11.6) |
| Age at first visit (years) |  |
| Mean (SD) | 38.3 (12.3) |
| Time between disease onset and first visit (months) |  |
| Median (IQR) | 22.1 (5.5-70.7) |
| Number of. of symptoms at onset - No. (%) |  |
| 1 | 4,542 (83.5) |
| ≥2 | 777 (14.3) |
| Symptoms at onset^a^ - No. (%) |  |
| Brain system | 1,344 (24.7) |
| Optic | 1,346 (24.8) |
| Supratentorial | 1,472 (27.1) |
| Spinal | 1,607 (29.6) |
| Age at diagnosis (years) |  |
| Mean (SD) | 36.3 (18.8) |
| Time between disease onset and diagnosis (months) |  |
| Median (IQR) | 7.1 (2.0-24.4) |
| First disease phenotype - No. (%) |  |
| Clinically isolated syndrome | 5,160 (94.9) |
| Primary progressive | 277 (5.1) |
| Disease phenotype at first visit^b^ - No. (%) |  |
| Clinically isolated syndrome | 1,409 (25.9) |
| Relapsing-remitting | 3,642 (67.0) |
| Primary progressive | 236 (4.3) |
| Progressive-relapsing | 41 (0.8) |
| Secondary progressive | 109 (2.0) |
| Type of DMT at first visit - No. (%) |  |
| No treatment | 2482 (45.6) |
| First-line treatment | 2,141 (39.4) |
| Second-line treatment | 669 (12.3) |
| Off-Label treatment | 146 (2.7) |
| EDSS score at first visit |  |
| Median (IQR) | 1.5 (1.0-2.5) |
| Multiple sclerosis disease severity at first visit |  |
| Median (IQR) | 3.7 (1.6-6.3) |
| Area of first visit - No. (%) |  |
| North-West | 1,583 (29.1) |
| North-East | 307 (5.7) |
| Center | 1,210 (22.3) |
| South | 1,602 (29.5) |
| Islands | 735 (13.5) |
| Deprivation index (quintiles) - No. (%) |  |
| I-II | 2,291 (42.1) |
| III | 539 (9.9) |
| IV-V | 2,607 (48.0) |
|  |  |

DMT: disease-modifying therapy; EDSS: Expanded disability status scale; IQR: interquartile range; SD: standard deviation. ^a^ Not mutually exclusive. ^b^ Defined as the last of the three disease courses recorded before the first visit to a center on the Register.

**Supplementary Table 2.** Characteristics of 106 centers included in the Italian MS and Related Disorders Register, 2018.

| **Characteristics** | **Centers** |
| --- | --- |
| Area of the center - No. (%) |  |
| North-West | 36 (34.0) |
| North-East | 14 (13.2) |
| Center | 22 (20.8) |
| South | 22 (20.8) |
| Islands | 12 (11.3) |
| Deprivation index (quintiles) - No. (%) |  |
| I-II | 39 (36.8) |
| III | 26 (24.5) |
| IV-V | 41 (38.7) |
| Type of center - No. (%) |  |
| Azienda Ospedaliera (Hospital)/Azienda Ospedaliera Universitaria (University Hospital) | 68 (64.2) |
| Presidio Ospedaliero ASL | 26 (24.5) |
| Other | 12 (11.3) |
| Organizational structure of the operating unit ^a^ - No. (%) |  |
| Complex | 72 (67.9) |
| Simple | 28 (26.4) |
| Other | 5 (4.7) |
| Center provides days or time dedicated to multiple sclerosis patients - No. (%) |  |
| No | 2 (1.9) |
| Yes | 104 (98.1) |
| Center with difficulties in access to DMT ^a^ - No. (%) |  |
| No | 58 (54.7) |
| Yes | 46 (43.4) |
| Hospital beds dedicated to multiple sclerosis patients - No. (%) |  |
| No | 66 (62.3) |
| Yes | 40 (37.7) |
| No. patients with multiple sclerosis followed at the center |  |
| Median (IQR) | 500 (250-1000) |
| Number of neurologists dedicated to multiple sclerosis |  |
| Median (IQR) | 3 (2-5.5) |
| Number of nurses dedicated to multiple sclerosis |  |
| Median (IQR) | 2 (2-4) |
| Presence of a PDTA ^a^ - No. (%) |  |
| No | 28 (26.67) |
| Yes | 77 (73.33) |
| Presence of a formal PDTA ^a^ - N. (%) |  |
| No | 63 (60.0) |
| Yes | 42 (40.0) |

DMT: disease modifying therapy; IQR: interquartile range; PDTA: Diagnostic and Critical Pathways; SD: standard deviation. ^a^ Discrepancies in the total are due to missing values.

**Supplementary Table 3.** Univariate odds ratios (OR) and corresponding confidence intervals (CI) for clinically isolated syndrome (CIS), primary progressive (PP)/progressive-relapsing (PR), and secondary progressive (SP) phenotypes at first visit compared to Relapsing-remitting (RR) phenotypes according to selected patients’ and center characteristics among 35,243 patients in the Italian MS and Related Disorders Register, 2000-2021.

|  | **CIS (9 310)** | **PP/PR (1 770)** | **SP (575)** |
| --- | --- | --- | --- |
|  | **OR (95% CI) ^a^** | **OR (95% CI) ^a^** | **OR (95% CI) ^a^** |
|  |  |  |  |
| Patients’ characteristics |  |  |  |
| Calendar year at first visit |  |  |  |
| 2000-2009 | 1^b^ | 1^b^ | 1^b^ |
| 2010-2014 | **0.88 (0.83-0.95)** | 1.00 (0.88-1.14) | **1.57 (1.26-1.97)** |
| 2015-2021 | 0.94 (0.88-1.00) | **1.21 (1.07-1.37)** | 1.22 (0.97-1.53) |
| Sex |  |  |  |
| Male | 1^b^ | 1^b^ | 1^b^ |
| Female | 0.99 (0.94-1.04) | **0.50 (0.45-0.55)** | **0.68 (0.57-0.80)** |
| Age at first visit |  |  |  |
| <35 years | 1^b^ | 1^b^ | 1^b^ |
| ≥35 years | **0.84 (0.80-0.88)** | **9.72 (8.25-11.45)** | **7.35 (5.69-9.50)** |
| Time between disease onset and first visit |  |  |  |
| <13 months | 1^b^ | 1^b^ | 1^b^ |
| ≥13 months | **0.11 (0.11-0.12)** | **2.39 (2.12-2.69)** | **15.27 (10.04-23.22)** |
| Center characteristics |  |  |  |
| Area of first visit |  |  |  |
| North-West | 1^b^ | 1^b^ | 1^b^ |
| North-East | **1.77 (1.03-3.02)** | 1.43 (0.87-2.34) | 1.39 (0.76-2.53) |
| Center | **1.62 (1.01-2.60)** | 1.06 (0.68-1.65) | 1.36 (0.79-2.33) |
| South | 1.54 (0.97-2.46) | 1.27 (0.83-1.96) | 1.51 (0.90-2.54) |
| Islands | 0.73 (0.40-1.34) | 1.24 (0.71-2.16) | 0.83 (0.42-1.67) |
| Deprivation index |  |  |  |
| I-II | 1^b^ | 1^b^ | 1^b^ |
| III | **0.59 (0.37-0.93)** | 0.81 (0.52-1.24) | 1.36 (0.80-2.31) |
| IV-V | 0.76 (0.51-1.13) | 0.93 (0.65-1.32) | 1.40 (0.90-2.18) |
| Number of patients with multiple sclerosis followed at the center |  |  |  |
| <500 | 1^b^ | 1^b^ | 1^b^ |
| ≥500 | 0.87 (0.61-1.23) | 0.98 (0.70-1.36) | 0.71 (0.48-1.06) |
| Number of neurologists dedicated to multiple sclerosis |  |  |  |
| <3 | 1^b^ | 1^b^ | 1^b^ |
| ≥3 | 1.12 (0.75-1.66) | 1.06 (0.73-1.54) | 0.84 (0.53-1.32) |
| Number of nurses dedicated to multiple sclerosis |  |  |  |
| <2 | 1^b^ | 1^b^ | 1^b^ |
| ≥2 | 1.20 (0.71-2.02) | 1.42 (0.80-2.50) | 0.95 (0.48-1.92) |
| Center with difficulties in access to DMT |  |  |  |
| No | 1^b^ | 1^b^ | 1^b^ |
| Yes | 1.37 (0.96-1.94) | 1.30 (0.95-1.79) | 1.14 (0.768-1.68) |
| Hospital beds dedicated to multiple sclerosis patients |  |  |  |
| No | 1^b^ | 1^b^ | 1^b^ |
| Yes | 0.86 (0.59-1.23) | 0.88 (0.63-1.23) | 1.09 (0.73-1.64) |
| PDTA |  |  |  |
| No | 1^b^ | 1^b^ | 1^b^ |
| Yes | 1.15 (0.76-1.72) | 1.08 (0.74-1.57) | 0.66 (0.43-1.03) |
|  |  |  |  |

DMT: disease-modifying therapy; PDTA: Diagnostic and Critical Pathways. ^a^ Odds ratio from multinomial, multilevel logistic regression models with random effects for center compared to RR phenotype. ^b^ Reference category.
